# Randomized Trial of a High Protein, Partial Meal Replacement Program with or without Alternate Day Fasting: Similar Effects on Weight Loss, Retention Status, Nutritional, Metabolic, and Behavioral Outcomes

**DOI:** 10.3390/nu10091145

**Published:** 2018-08-23

**Authors:** Jane Bowen, Emily Brindal, Genevieve James-Martin, Manny Noakes

**Affiliations:** CSIRO Health and Biosecurity, Adelaide, SA 5000, Australia; emily.brindal@csiro.au (E.B.); Genevieve.James-Martin@csiro.au (G.J.-M.); manny.noakes@csiro.au (M.N.)

**Keywords:** weight loss, fasting, meal replacement

## Abstract

Higher-protein diets, meal replacements, and greater early weight loss have separately been associated with greater weight loss. We compared a high-protein, meal replacement program with daily energy restriction (DER) to one which provided greater energy restriction adding alternate day fasting (ADF + DER; alternating days of modified-fasting and DER plus 1 *ad libitum* day/week) on retention, weight loss, physiological, nutritional, and behavioral markers. Participants were randomized to ADF + DER or DER for 16 weeks (*n* = 162, age 40 ± 8 years BMI 36 ± 6 kg/m^2^ (Mean ± SD)) plus 8 weeks weight maintenance. At week 16 weight change was −10.7 ± 0.5 kg and −11.2 ± 0.6 kg in ADF + DER and DER groups (treatment NS). Fat mass, visceral adipose tissue, and lean mass (*p* < 0.05) were similarly reduced between treatments. Weight loss was sustained to 24 weeks (treatment NS). Fasting LDL-cholesterol, triglycerides, insulin, hsCRP, glucose, and blood pressure all improved (*p* < 0.05; treatment NS). Transferrin saturation, ferritin, serum zinc, folate, and B12 improved (*p* < 0.05; treatment NS). Plasma thiamine and vitamin D levels decreased, reflecting lower carbohydrate intakes and seasonal changes, respectively. Food cravings, quality of life, and mood improved (treatment NS). Energy, fatigue, and pain improved slightly more in DER (*p* < 0.05). This study supports the use of higher protein, meal replacement programs with or without ADF in weight management.

## 1. Introduction

Compliance with, and long-term adherence to, traditional continuous daily energy restricted (DER) weight loss diets is poor, and adherence is often affected by psychological and behavioral factors associated with interventions [1]. Motivation to adhere to weight loss programs can also be diminished by a slow rate of weight loss [2]. However, there is evidence that some characteristics of weight management approaches may result in more effective medium to longer term weight loss outcomes. Both higher protein dietary patterns [3] and meal replacement programs [4] have each been assessed separately in randomized controlled trials. In addition to these characteristics, greater early weight loss has also been associated with greater longer term weight loss [5] but has not been prospectively assessed in randomized controlled trials. Higher protein dietary patterns have evidence to support both appetite control, differential improvements in body composition, as well as longer term weight loss maintenance [6,7]. Adults with overweight/obesity also find meal replacement programs (MRP) for weight loss easier to follow [8] as initial adherence is aided by their simple structure and novelty, relative to an individual’s usual eating pattern. Successful early weight loss at 1–2 months has been associated with weight loss at 8-years [5], although whether early accelerated weight loss is beneficial has not been prospectively assessed. We have previously reported on outcomes of a higher protein MRP [9] and investigated variations that may assist with weight loss which may enhance longer term adherence.

Intermittent energy restriction such as alternate day modified fasting (ADF) is another strategy for weight loss that has gained scientific interest. This approach alternates between a highly restricted calorie intake 3–4 days per week, with days of eating freely and to appetite (*ad libitum*). Systematic reviews comparing ADF to daily energy restriction (DER) have concluded that DER and ADF are equivalent in terms of short- and longer-term weight loss [10,11]. Meta-analysis by Harris et al. [12] showed both treatment interventions achieved similar changes in body weight of approximately 7 kg and that ADF approaches in study durations of more than 12 weeks were more effective than no treatment for weight loss (−4.14 kg; 95% CI −6.30 kg to −1.99 kg; *p* ≤ 0.001). However, it has been noted that interpretation of the ADF literature has been limited by small sample sizes [13].

Improved satiety, increased protein intake, and better adherence to fast-day calorie goals have shown to be characteristics of participants who achieve clinically significant weight loss with ADF [14]. There are no reports on the impact of ADF approaches on objective measures of nutritional status. The majority of ADF protocols in the literature have compared ADF isocalorically to DER. This is appropriate to understand the differential physiological and metabolic effects independent of weight loss. However no studies to date have intentionally evaluated ADF as a strategy to augment weight loss through greater energy restriction, in order to prospectively assess if accelerated early weight loss results in better retention and weight loss outcomes.

Whilst the literature on ADF is growing, there are several opportunities to also extend the literature using what is known about successful weight loss strategies. Assessing whether a planned greater energy restriction through the use of ADF in combination with higher dietary protein composition and meal replacements has physiological and behavioral advantages over a similar program without ADF is not known. To our knowledge, the combination of DER plus ADF has not previously been compared to DER alone.

Therefore, the aim of this study was to compare two high protein meal replacement programs—one which provides the same daily energy restriction (DER) and the other which further restricts kilojoules through alternate energy restriction; 3 days of ADF, 3 days of alternate DER, and one *ad libitum* day (ADF + DER). We aimed to comprehensively assess these two dietary approaches on participant retention, weight loss, and body composition in adults with overweight/obesity. A secondary aim was to compare the impact of these higher protein weight loss strategies on markers of metabolic health, nutritional status, quality of life, and eating behaviors. Finally, this study assessed maintenance of weight loss from week 16 to 24.

We hypothesized that the ADF + DER would be associated with greater overall retention, weight loss and weight loss maintenance, and metabolic improvements compared to DER.

## 2. Materials and Methods

The trial was registered with the Australian New Zealand Clinical Trials Registry (http://www.anzctr.org.au ACTRN12616000110482) and approved by the CSIRO (Commonwealth Scientific and Industrial Research Organization) Human Research Ethics Committee (05/2015).

### 2.1. Participants

Adults with overweight/obesity (aged 25–60 years; Body Mass Index (BMI) >27.0 kg/m^2^) were recruited via public advertisement and participated in this single-center, randomized, controlled trial conducted at the CSIRO Clinical Research Unit in Adelaide, Australia between February and September 2016. Sample size calculations were based on the expected proportion retained in the two treatment groups using the *z* family of tests (proportions; G*power). Eighty-two participants per group provided 80% power to detect an increase in the proportion of the sample retained from 60% in the DER to 80% in the ADF + DER (*α* = 0.05) based on our previous study [9].

Exclusion criteria were pre-existing diagnosed medical conditions (including type 1 or 2 diabetes), any significant endocrinopathy (other than stable, treated thyroid disease); history of bariatric surgery; malignancy (other than non-melanoma); liver, respiratory, renal, gastrointestinal or cardiovascular disease; pregnancy or lactation; epilepsy; clinical depression; history/risk of (score of ≥2 on the SCOFF screening questionnaire for eating disorders [15]) or current eating disorder; food allergy to the prescribed foods; or smoking.

Eligible participants attended an information session and provided written, informed consent to the study protocol.

### 2.2. Study Design

This parallel study comprised of 16 weeks adhering to one of two energy restricted dietary protocols, followed by 8 weeks of a weight maintenance dietary protocol. Participants were block matched for age, sex, and BMI, and assigned to either daily continuous energy restriction meal replacement program (DER) or modified, alternate day fasting meal replacement program (ADF + DER) in a 1:1 ratio using a random sequence (generated via www.randomization.com). Randomization procedures were performed by researchers who were independent of delivering the intervention and assessing outcomes. Participants and research dietitians delivering intervention content could not be blinded, however the remaining study team were blinded to the randomization. Data analysis was undertaken prior to breaking the randomization code.

At baseline participants attended the research clinic after an overnight fast for assessment of all outcomes, including collection of blood samples. Participants then attended every two weeks individual appointments with a research dietitian to measure body weight, blood pressure (weeks 2, 4, 8, and 12), assess dietary compliance, and collect meal replacements. During week 16, participants repeated the same outcome assessments as at baseline and advice was provided for switching to weight maintenance dietary protocol. At week 24, body weight was measured and behavioral questionnaires were completed on a computer in the clinic.

### 2.3. Dietary Intervention Protocol

The dietary protocols are summarized in Table 1. The DER intervention involved a daily energy restriction regimen of one meal and a prescribed number of meal replacements and snacks between meals. The ADF + DER alternated between the DER (Tuesday, Thursday, and Sunday), a modified fasting regimen (Monday, Wednesday, and Friday) and one day per week to eat *ad libitum* (Saturday). In addition, the schedule was designed to maximize the duration between the *ad libitum* day and the week 16 blood collection for biochemical analysis, which took place on a weekday.

Trained research dietitians delivered and monitored the dietary intervention for both groups. Individual estimated energy requirements were calculated for all participants at study commencement [16] based on actual body weight, multiplied by appropriate physical activity levels and reduced by 30% to achieve energy restriction for weight loss. This individualized energy level determined the number of formulated meal replacements and the number of permitted snacks for each participant. One adjustment to the dietary protocol (increase or decrease of one meal replacement or snack per day) was permitted in week 2, if the participant reported difficulty managing the dietary protocol. After this time, no further dietary changes were allowed.

Meal replacements were provided to all participants during the energy restriction phase at no cost (Impromy^TM^, manufactured by Probiotec Pty Ltd., Laverton North, Australia). Meal replacements were available in three flavors (vanilla, chocolate, and latte) and required reconstitution with 250 mL of either skim milk or a dairy-free alternative (unsweetened, calcium-enriched). The ‘as consumed’ nutrient composition of the meal replacements was approximately 1000 kJ, 25 g protein, 4 g fat, 27 g carbohydrate, and 6 g fiber with each containing 25% recommended daily intake for Vitamin A, Thiamin, Riboflavin, Niacin, Folate, Vitamin B6, Vitamin B12, Vitamin C, Vitamin D, Vitamin E, Calcium, Iodine, Iron, Magnesium, Phosphorus, and Zinc.

The main meal for the DER diet comprised of lean meat, poultry, or fish (200 g raw weight), 2 cups of specified low kilojoule salad/vegetables and 10 g oil, providing a total of 1.5–2 MJ and 40–45 g protein. Guidelines and recipes for this meal were provided. Participants were also given a list of permitted between-meal snacks (~500 kJ portions) based on fruit, low-fat dairy, wholegrains, and nut/seed/legume options. All participants were encouraged to drink water and were advised of optional vegetables, beverages, and condiments with minimal kilojoule content that could be consumed as needed, to manage hunger.

Participants allocated to the ADF + DER group followed the DER program for three set days per week (Monday, Wednesday, and Friday) and alternated with three set modified fasting days (Tuesday, Thursday, and Sunday) as described above. Food allowance on the modified fasting days was limited to the individual’s allocated number of meal replacements and a meal comprising of low energy salad/vegetables only. Guidelines and recipes for preparing this meal were provided. No in-between-meal snacks were permitted on the modified fasting days. On the *ad libitum* day participants were advised that they could eat to appetite, choosing the type and quantity of food and beverages; intake was not recorded. Based on an estimated average energy intake on the *ad libitum* day of 10 MJ, it was projected that the ADF + DER could have an average net additional energy deficit of approximately −700 kJ/day, compared to the DER. If maintained over 16 weeks, this was estimated to result in an additional −2.8 kg of weight loss in the ADF + DER group.

Planned energy and macronutrient composition of the DER diet was: 31% of total energy as carbohydrate, 38% protein, and 28% total fat (52% monounsaturated fat and 17% polyunsaturated fat). Planned energy and macronutrient composition of the modified fasting days was: 40% carbohydrate, 34% protein, and 22% total fat (55% monounsaturated fat and 9% polyunsaturated fat).

Participants met individually with a study dietitian every two weeks until week 16. These visits included: troubleshooting, a review of dietary compliance which was assessed by a self-completed 14-day checklist, and a self-rated assessment of compliance to the dietary program on a scale out of 5 stars (where 1 star represented not following the diet at all and 5 stars represented following the diet completely). At week 16 participants in both groups were advised by the dietitians to switch to a weight maintenance diet for a further 8 weeks. Individualized guidance on a higher protein, food-based (i.e., no meal replacements) dietary protocol was tailored to each individuals’ energy requirement for weight maintenance using a units-based approach [17]. Both groups were advised to follow the same weight maintenance diet.

Participants were recommended to continue with their habitual exercise patterns; no specific advice for exercise was provided and exercise was not recorded.

### 2.4. Study Outcomes

Outcomes were assessed in week 0 and 16. Primary outcomes were participant retention (calculated as the percentage of participants in each treatment group attending the scheduled clinic visit) and change in body weight. Body weight was measured at all clinic visits using calibrated electronic digital scales (Mercury, AMZ 14, Tokyo, Japan). BMI was calculated using the formula: weight (kg)/height (m^2^). Height was measured using a stadiometer (SECA, Hamburg, Germany). Dual Energy X-ray Absorptiometry (DEXA; Lunar Prodigy; General Electric Corporation, Madison, WI, USA) was used to measure body composition; total fat mass (FM) and fat-free mass (FFM).

Seated blood pressure was measured in triplicate at week 0, 2, 4, 8, 12, and 16 by automated sphygmomanometry (SureSigns VS3, Philips, Andover, MA, USA).

Fasting blood samples were collected, centrifuged at 3500 rpm at 4 °C for 15 min (Beckman Coulter Allegra X-12R), and the supernatant was stored at −80 °C. After study completion, plasma glucose, serum total cholesterol, HDL-C, TG, and high sensitivity C-reactive protein (hsCRP) were measured on a Beckman Coulter AU480 Chemistry Analyzer (Beckman Coulter Inc., Brea, CA, USA) using standard enzymatic kits for glucose and lipids and immuno-turbidimetric test for hsCRP (Beckman Coulter Inc., CA, USA). LDL-C levels were calculated by the Friedewald equation [18]. Plasma insulin concentration was measured using a commercial enzyme immunoassay kits (Mercodia AB, Uppsala, Sweden). Plasma iron studies, red cell folate, thiamine, 25 hydroxy vitamin D, serum zinc, and vitamin B12 analyses were performed at a commercial laboratory (SA Pathology, Adelaide, Australia).

Behavioral parameters were collected at weeks 0 and 16 via a computer-assisted survey in the clinic. This included assessment of food cravings using the 21-item Trait Food Craving Questionnaire [19], motivation and behavioral control to stay on diet using seven questions, designed based on the Theory of Planned Behavior [20], mood using the validated 20-item Positive and Negative Affect Schedule [21] and health-related quality of life using the MOS SF-36 [22].

### 2.5. Statistical Analysis

Non-normally distributed data were logarithmically transformed prior to analysis (i.e., serum hsCRP, insulin, and ferritin). Data were screened for univariate and multivariate outliers. Sensitivity analyses were performed for models with and without outliers and due to negligible effects on final outcomes, outlying values were retained in the interest of retaining complete data. Group differences in baseline characteristics were compared using independent *t*-tests.

The primary analysis to assess differential responses between groups were performed using mixed effects linear models. Each participant was treated as a fixed effect in the repeated measures model, allowing each participant to have their own intercept and slopes that enables more precise modelling of longitudinal changes. The model included all available data from the 163 participants who commenced the intervention, and an unstructured repeated covariance matrix. Treatment and time were included as fixed factors and analyzed for main effects and treatment × time interaction effects.

Statistical analysis was performed using SPSS software version 23 (IBM Corporation, New York, NY, USA). Results are presented as means and standard deviations (SD) of the raw data. Statistical tests were two-tailed with statistical significance set at *p* < 0.05.

## 3. Results

### 3.1. Participants and Retention

**Figure 1:**
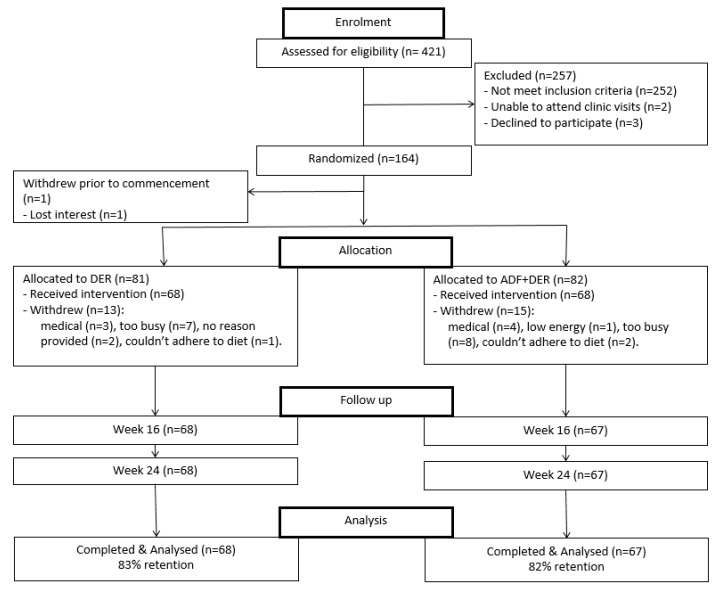
CONSORT diagram indicating sample size at each stage of the study. DER, standard meal replacement program; ADF + DER modified meal replacement program.

Four-hundred-and-twenty-one participants were assessed for eligibility and 164 were randomized to the two treatment groups. Participant retention was 83% and 82% at week 16 for the DER and ADF + DER, respectively (Figure 1). There was no difference between treatments (*p* > 0.05). The same number of participants were retained up until week 24. Baseline characteristics of participants did not differ significantly between treatment groups (Table 2).

### 3.2. Body Weight, Composition, and Metabolic Markers

**Figure 2:**
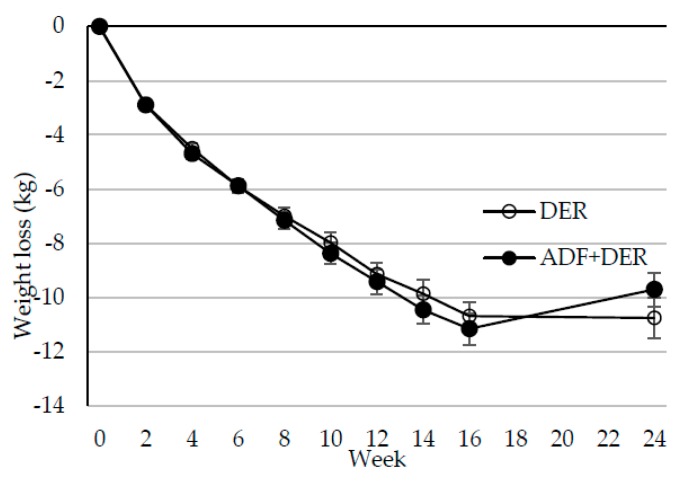
Mean ± standard error change in bodyweight after 16 weeks of the DER (*n* = 68) or ADF + DER diet (*n* = 67), followed by eight weeks of weight maintenance. DER, standard meal replacement program; ADF + DER modified meal replacement program. For between group differences, *p* > 0.05; for time effect *p* < 0.01; for group differences over time (time × diet interaction) *p* > 0.05, using linear mixed-effects model analysis.

Figure 2 shows the trajectory of weight loss over the 24 weeks by dietary intervention. Results from the mixed models indicated a significant reduction in body weight over time (*p* < 0.001) with no significant weight change between weeks 16 to 24. At week 16, weight change from baseline was −10.7 ± 0.5 kg in the ADF + DER group and −11.2 ± 0.6 kg in the DER group (Table 3). There was no significant treatment or time by treatment interaction for body weight.

At week 16, there was a significant reduction in fat mass, visceral adipose tissue, and lean mass (*p* < 0.05), with no treatment or time by treatment interactions (Table 3). There were significant reductions in fasting total cholesterol, LDL-cholesterol, HDL-cholesterol, triglycerides, hsCRP, insulin, glucose, and systolic and diastolic blood pressure at week 16 compared to baseline (*p* < 0.05), independent of treatment (Table 3).

### 3.3. Nutritional Markers and Reported Compliance with Dietary Protocol

There was a significant increase in plasma transferrin saturation, ferritin, folate, vitamin B12, and zinc at week 16 (*p* < 0.05), independent of treatment (Table 4). There was a significant reduction in thiamine, transferrin, and vitamin D in both groups by week 16 (*p* < 0.05).

**Figure 3:**
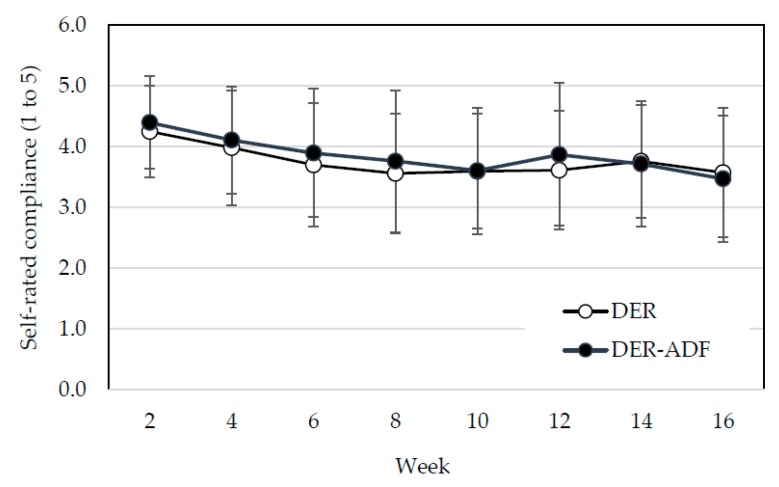
Self-rated compliance (out of five stars) to dietary program for DER and ADF + DER groups. DER, standard meal replacement program; ADF + DER modified meal replacement program. For between-group differences, *p* > 0.05; for time effect, falls in compliance were significant between week 2 and all other visits (*p* < 0.05 to *p* < 0.001) for both groups; for group differences over time (time × diet interaction) *p* > 0.05, using linear mixed-effects model analysis.

There were significant changes in self-rated compliance to the diet program over time (*p* < 0.001) independent of treatment. There were no interaction effects. Falls in compliance were significant between visit 2 (week 2) and all other visits (*p* < 0.05 to *p* < 0.001) for both groups (Figure 3).

### 3.4. Behavioral Measures

#### 3.4.1. Food Cravings

**Figure 4:**
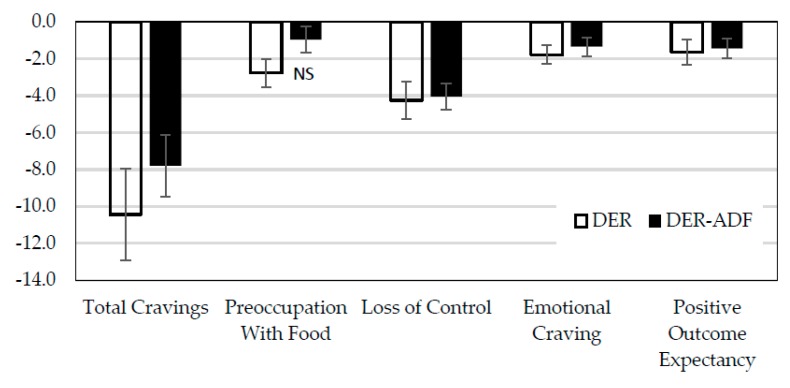
Food Craving scores for those in the DER and ADF + DER groups. Note: scores based on Trait Food Craving Questionnaire: Total caving scores range min 21, max 126; Preoccupation with Food min 6, max 36; Loss of Control min 6, max 36; Emotional Craving min 4, max 24; Positive Outcome Expectancy min 5, max 30. DER, (*n* = 68) standard meal replacement program; ADF + DER (*n* = 67) modified meal replacement program; NS, not significant. For between-group differences, *p* > 0.05; for time effect falls in total cravings, loss of control, emotional cravings, and positive outcome expectancy, *p* < 0.0001; an interaction effect revealed a fall in preoccupation with food only for the DER group (*p* < 0.05), using linear mixed-effects model analysis.

There were significant decreases in total craving level, and for subscales of loss of control, emotional cravings and positive outcome expectancies (all *p* < 0.001) at week 16 compared to baseline, independent of treatment. There was a significant interaction effect for preoccupation with food (*p* < 0.05). Post-hoc analyses revealed that the change in this outcome was only significant for those in the DER group (Figure 4).

#### 3.4.2. Changes in Health-Related Quality of Life

**Figure 5:**
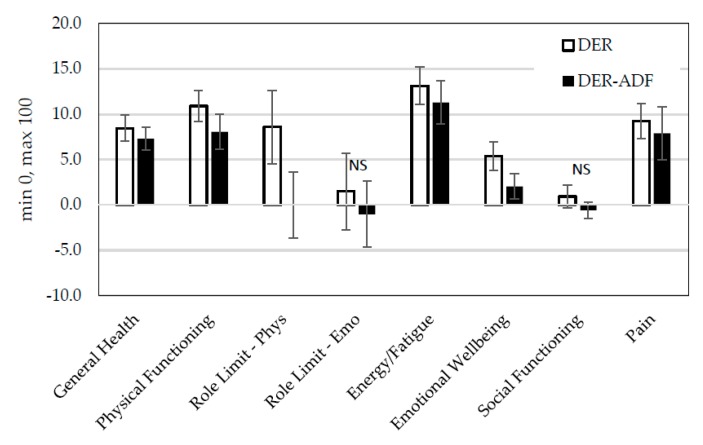
Changes in health-related quality of life scores (MOS SF36) between baseline and week 16 presented by treatment. DER, (*n* = 68) standard meal replacement program; ADF + DER (*n* = 67) modified meal replacement program; Phys, Physical; Emo, Emotional; NS, not significant. Note: Scores out of a possible 100 with higher scores representing improved health-related quality of life. For between-group differences, *p* > 0.05; for time effect improvements in general health, physical functioning, energy, emotional wellbeing, pain *p* < 0.0001; an interaction effect revealed an improvement in role limitations due to physical function only for the DER group (*p* < 0.05), using linear mixed-effects model analysis.

Emotional wellbeing, general health, physical functioning, energy/fatigue, and pain (all *p* < 0.001) all improved over 16 weeks, regardless of treatment. Social functioning and emotional role limitations did not differ by time or treatment or the interaction between the two (Figure 5).

For role limitations due to physical function there was a significant interaction (*p* < 0.05). At follow-up, the DER group had significantly higher scores (*p* < 0.05).

#### 3.4.3. Mood

**Figure 6:**
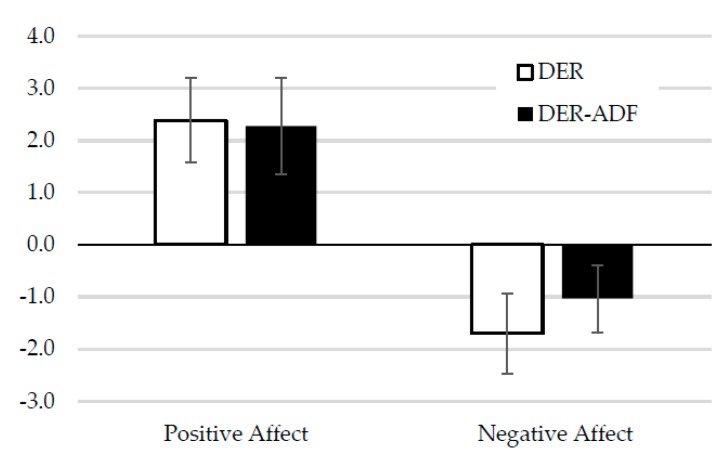
Changes in mood (positive and negative affect) presented by dietary allocation. Note: Scores based on the Positive and Negative Affect Schedule with a range from 10 to 50 with higher scores representing higher levels of positive or negative emotions. DER, (*n* = 68) standard meal replacement program; ADF + DER (*n* = 67) modified meal replacement program. For between-group differences, *p* > 0.05; for time effect *p* < 0.01 for decrease in negative affect and *p* < 0.001 for increase in positive affect; the interaction effect was *p* > 0.05, using linear mixed-effects model analysis.

There was a small, significant increase in positive affect (mood) (*p* < 0.001). This was accompanied by a significant decrease in negative affect (mood) (*p* < 0.01). There were no effects of treatment and no interactions (Figure 6).

## 4. Discussion

This study was intentionally aimed at comparing two similar high protein meal replacement programs that differed in kilojoule intake through adopting an alternate day fasting protocol in one of them. The rationale was based on data that has shown that larger initial weight losses are associated with greater net weight loss in the longer term [23,24] and that both higher protein dietary patterns [3] and meal replacement programs [4] have each been shown to achieve greater medium to longer term weight loss. Although our study was one of the largest and most comprehensive studies conducted to date on ADF, the combination of ADF with DER has not to our knowledge previously been tested.

We hypothesized that a lower kilojoule intake through the addition of ADF would result in greater early stage weight loss, improve retention, and achieve better longer-term weight loss and metabolic markers. This hypothesis was not able to be upheld as we found no weight loss differential between treatment groups.

We found that both ADF + DER and DER participants achieved a pooled mean weight loss of 11% over 16 weeks with an unanticipated finding of no group differences in weight loss or body composition including lean and fat mass changes. This was despite the planned kilojoule difference of 4900 kJ/week (−700 kJ/day) which would have resulted in an anticipated a weight loss of difference of approximately 2.8 kg over the 16-week active weight loss phase. There are several possible explanations to our observation. This difference was based on numerous assumptions including a projected estimate of what individuals might consume on the *ad libitum* day (10 MJ), the assumption that participants were compliant with their prescribed diet for the 16 week duration and that there were no adjustments to prescribed kilojoule intake over the course of the study. We also did not adjust for reductions in energy deficit during weight loss which may also have provided a lower weight loss difference estimate.

It is possible that ADF + DER participants overcompensated on their one *ad libitum* day such that the energy differential between groups was minimized. However, this has not been observed in prior ADF studies [12] and Clayton et al. [25] have shown that overcompensation does not occur in the subsequent 48 h after a 24 h severe energy restriction period. Alternatively, it may be possible that the consistently higher protein intake of the DER encouraged more consistent compliance and by contrast, less so in the ADF + DER group. However, this is not supported by our compliance data as those on ADF + DER had similar perceived compliance to those on DER. Perhaps a potentially plausible rationale may be related to both the higher protein intake and meal regularity of the DER. Meal irregularity is associated with a lower thermic effect of meals [26] as is a lower kilojoule intake. Higher protein dietary patterns have been reported to mitigate reductions in resting energy expenditure of 595.5 kJ/day compared to higher carbohydrate patterns [7]. Also meal regularity appears to be associated with greater thermic effect of food which may favor negative energy balance [26] and lower kilojoule intakes also have lower thermogenic effects [27]. The same is also true with higher protein meals which have a higher thermic effect [28,29,30] suggesting that there is an added energy-cost associated with a high-protein dietary pattern which may have reduced the planned energy differential between ADF + DER and DER participants. This is due to a 40–45 g/day lower protein intake three days per week on ADF + DER relative to DER. A prior study by Catenacci et al. [31] has also shown that despite a greater planned energy deficit (372 kCal) in the alternate day fasting group, there were no significant differences in weight loss to the continuous energy restriction group. The authors theorized the following, (1) the ADF group under-reported food intake on fast and/or fed days or (2) fasting led to a reduction in some component of nonresting energy expenditure in the ADF group (e.g., physical activity energy expenditure and thermic effect of food) and suggested that future studies might include more accurate assessments of energy intake and expenditure.

In general, intermittent fasting appears equally effective as daily energy restriction for short-term weight loss, with a 3–5 kg weight loss achieved after approximately 10 weeks [11]. However, there are many variations of intermittent fasting, including ADF, both in macronutrient composition, number, and severity of restriction on “fasting” days, the use of *ad libitum* days and the timing of meals. Some intermittent fasting protocols ensure that participants on fasting days abstain from food after the allocated morning meal. In our study, we incorporated an evening meal of low kilojoule vegetables which may have impacted appetite regulation differently than abstaining from food, irrespective of energy content. However, Hoddy et al. [32] have shown that meal timing on fasting days produced similar weight loss irrespective of whether food was eaten at breakfast, lunch or dinner.

Alhamdan et al. [33] compared ADF studies to very low calorie diets (VLCD) in a meta-analysis of 10 studies (four ADF and six matched VLCD). These comparisons were made irrespective of the different energy content of these approaches which would tend to be lower for the VLCD. Adjusting for BMI and duration, the authors found no significant difference in mean body weight loss between the diets. Of interest, fat-free mass loss was lower on ADF (VLCD 1.69 kg more fat-free mass loss than ADF) with a significant difference also observed in fat mass loss which favored ADF (ADF 3.31  kg more fat mass loss than VLCD). The conclusion was that ADF is an efficacious dietary method, and superior to VLCD for some patients because of ease of compliance, greater fat-mass loss, and relative preservation of fat-free mass. In randomized trials of ADF and continuous energy restriction (CER), Varady et al. [13] found that weight and fat mass loss was similar up to 12 weeks but that less fat-free mass was lost on ADF suggesting that ADF may be more effective for lean mass retention. We did not observe a significant difference in body composition changes in our study as the level of CER was not as great as VLCD and the higher protein content of CER and ADF treatments would be greater than a VLCD and mitigate against lean mass loss.

In terms of longer-term weight loss maintenance with intermittent fasting, there have been fewer studies. Headland et al. [34] undertook a systematic review and meta-analysis of six such studies lasting a minimum of six months in 2016, noting that there were no differences in the magnitude of weight loss relative to CER and that studies lasting >12 months were needed. However, there was only one ADF study included and it was also noted that the number of long-term studies conducted was very limited, participant numbers typically less than 50 and that the dietary protocols and supervision were widely diverse. Hence conclusions about intermittent fasting are not necessarily generalizable to all protocols including ADF.

Following the active weight loss phase, participants ceased meal replacements and were successfully able to maintain their weight on a higher protein whole food eating pattern for a further eight weeks with no professional supervision. This suggests that MR in the context of use in this study did not result in rebound weight gain, at least at the 6-month timeframe. A prior study using a high protein diet protocol for 12 weeks [35] incorporated energy restriction on a high protein diet for six days and fasting for one day. The average loss of body weight reached 10% but when assigned to weight loss maintenance on either a higher carbohydrate diet or a higher protein diet, only the latter group was able to maintain weight loss. This is consistent with the outcome of our study. This is also consistent with Kroeger et al. [14] concluding that successful weight loss at 1 year on ADF was related to increased protein intake. Our observation that craving scores decreased during both interventions is notable and may have contributed to the lack of rebound weight gain. While participants were prescribed a higher protein diet for weight maintenance, a limitation of the current study is that we did not assess whether participants from either group independently chose to incorporate ADF or meal replacements to assist with weight maintenance.

Metabolic markers similarly and substantially improved in both groups as did body composition which showed a net FM loss of 8 kg and a FFM loss of 1.65 kg, comparable to other high protein diet interventions [7].

Markers of nutritional status have rarely been assessed in prior ADF or MR studies. Noakes et al. [8] compared a partial MR program to CER with whole foods and showed that serum folate and plasma beta-carotene were higher in the MR group, suggesting a higher vegetable intake in the MR group. Raynor et al. [36] demonstrated that a partial meal replacement plan consumed by Intensive Lifestyle Intervention in the Action for Health in Diabetes (Look AHEAD) trial participants was related to superior diet quality at one year. Diet quality was assessed subjectively through a food frequency questionnaire. Our study objectively measured biological markers of nutritional status, demonstrating that both MR programs showed improved nutritional status in several markers over the active weight loss period. One notable exception was thiamine status, which decreased similarly in both groups. Although the meal replacements provided at least 50% of the RDI for thiamine when replacing two meals, it is likely that the meal patterns which were low in thiamine containing carbohydrate foods may have contributed to the decline. The Recommended Daily Intake for thiamine for men is 1.2 mg/day, and 1.0 mg/day for women. Each MR provided 0.3 mg of thiamine. We have previously shown that during weight loss in type 2 diabetes, erythrocyte thiamine pyrophosphate (TPP) decreased on a diet with adequate thiamine (1.1 mg/day) but remained unchanged on a high thiamine diet (2.8 mg/day) [37]. Whether attention to thiamine status may be required in MR programs is conjectural; the relevance of this change may not have clinical implications as participants’ thiamine status remained within the normal range. However, clinical thiamine deficiency has been shown to be common in individuals with medically complicated obesity and in pre-operative obesity surgery patients [38,39].

Vitamin D status declined only marginally due to seasonal changes suggesting that the vitamin D composition of the MR may have mitigated greater reductions. There was a small reduction in plasma transferrin at week 16 in both groups compared to baseline (*p* < 0.05) but not to transferrin saturation. These levels were maintained within the normal ranges and hence unlikely to have any clinical relevance for this study population.

As we aimed to assess the experiences of participants on either program, we undertook a series of behavioral measures. Self-rated compliance was generally high on both treatments. We observed reductions in food cravings and increases in mood independent of treatment. Food cravings reductions are known to consistently decline with energy restriction and weight loss [40] as specifically also VLCD MR programs [41]. Measures of health-related quality of life improved equally on both treatments. Previous assessment of groups with minor versus serious medical conditions revealed scores between 15–20 points depending on the subscale [42]. For example, those with serious medical or psychiatric conditions had energy scores 15 points lower than those with only minor medical conditions. Therefore, an improvement of 10 represents a significant improvement in this outcome. Health-related quality of life improvements are not consistently associated with weight loss [43,44] and the nature of the intervention and study populations may play a role in this discrepancy.

Limitations of this trial include the lack of biological data at six months, lack of longer term follow up and the limited numbers of male participants which may limit generalizability of these findings. We were also unable to comprehensively assess dietary intake as this would have imposed potential dietary restraint for the *ad libitum* day in the ADF + DER group. Similarly we did not assess dietary intake after the 16 week intervention and to what extent participants continued MR and/or fasting days. However, this period may be more reflective of free living conditions and demonstrated that weight loss was sustained to six months. Given the planned differences in energy intake, a further limitation was the lack of measures of energy expenditure and physical activity monitoring. In the future, more accurate assessments of energy intake and expenditure in such studies may be warranted. Strengths include the large sample size, comprehensive behavioral, anthropometric and biological markers, and the similarity of the two dietary interventions which allow an objective assessment of the alternate day fasting approach.

## 5. Conclusions

Both high protein ADF + DER and DER programs were well-accepted, effective in achieving improved metabolic and nutritional markers as well as sustained 10% weight loss to six months. These changes were also accompanied by improved mood, lower food cravings, reduced pain, and greater quality of life. Collectively, this study supports the use of higher protein, partial meal replacement programs with or without alternate day fasting in weight management.

## Figures and Tables

**Table 1:** Summary of the continuous energy restriction meal replacement program ^a^ (DER) and alternate day fasting meal replacement program (ADF + DER) dietary protocols.

	DER	ADF + DER		
	Mon-Sun	M,W,F	Tu,Th,Su	Sa
Breakfast	MR ^b^	MR ^b^	MR ^b^	*ad libitum*
Snack ^c^	1 snack	1 snack	-	
Lunch	MR ^b^	MR ^b^	MR ^b^	
Snack ^c^	1 snack	1 snack	-	
Dinner	200 g lean protein + low energy vegetables + 2 teaspoons oil	200 g lean protein + low energy vegetables + 2 teaspoons oil	Low energy vegetables	
Energy (kJ)	5000	5000	2400	10,000 (estimate)
Protein (g)	102	102	55	NA

DER, continuous energy restriction meal replacement program; ADF + DER, alternate day fasting meal replacement program; MR, meal replacement; M, Monday; Tu, Tuesday; W, Wednesday; Th, Thursday; F, Friday; Sa, Saturday; Su, Sunday; NA, not assessed. ^a^ The total daily macronutrient distribution for the DER diet shown above was 31% of total energy as carbohydrate, 38% protein, and 28% total fat (52% monounsaturated fat and 17% polyunsaturated fat). ^b^ Impromy^TM^, manufactured by Probiotec Pty Ltd., Laverton North, Australia; MR was ~1000 kJ, 25 g protein, 4 g fat, 27 g carbohydrate, 6 g fiber with each containing 25% recommended daily intake for Vitamin A, Thiamin, Riboflavin, Niacin, Folate, Vitamin B6, Vitamin B12, Vitamin C, Vitamin D, Vitamin E, Calcium, Iodine, Iron, Magnesium, Phosphorus, and Zinc. The number of meal replacements allocated per day was determined by individual energy requirements for weight loss. ^c^ Prescribed snack options: fruit, low fat dairy, wholegrains, and nut/seed/legume. The number of snacks allocated per day was determined by individual energy requirements for weight loss.

**Table 2:** Baseline participant characteristics ^1^.

	DER (*n* = 81)	ADF + DER (*n* = 82)
Age (years)	40.6 ± 8.8	40.0 ± 8.3
Sex, Female *n* (%)	65 (80)	67 (82)
Male *n* (%)	16 (20)	15 (18)
Body weight (kg)	99.6 ± 15.6	100.6 ± 19.6
BMI (kg/m^2^)	35.5 ± 5.5	35.7 ± 5.8

^1^ Values are Means ± SD. Body weight assessed in fasting state. DER, standard meal replacement program; ADF + DER modified meal replacement program; BMI, body mass index. No significant differences between groups at baseline.

**Table 3:** Anthropometric and metabolic measurements in the DER and ADF + DER groups before and after the 16-week energy restriction intervention ^1^.

	DER		ADF + DER		*p*
	Baseline (*n* = 81)	Week 16 (*n* = 68)	Baseline (*n* = 82)	Week 16 (*n* = 67)	
Body weight ^2^, kg	99.6 ± 15.6	87.1 ± 14.0	100.6 ± 19.6	90.6 ± 18.7	0.803
BMI ^2^, kg/m^2^	35.5 ± 5.5	31.1 ± 5.1	35.7 ± 5.8	31.9 ± 5.5	0.913
Total FFM ^2^, kg	50.7 ± 7.9	48.8 ± 7.6	51.1 ± 9.1	49.7 ± 9.2	0.842
Total FM ^2^, kg	45.1 ± 11.2	34.8 ± 10.2	45.7 ± 13.1	37.3 ± 12.8	0.674
TC ^2^, mmol/L	5.3 ± 1.0	4.7 ± 0.9	5.2 ± 1.0	4.7 ± 1.0	0.754
Triglycerides ^2^, mmol/L	1.5 ± 0.8	1.1 ± 0.5	1.4 ± 0.6	1.2 ± 0.6	0.029 ^3^
HDL-C ^4^, mmol/L	1.3 ± 0.3	1.3 ±0.3	1.3 ± 0.3	1.2 ± 0.2	0.154
LDL-C ^2^, mmol/L	3.3 ± 0.9	2.9 ± 0.7	3.3 ± 0.9	2.9 ± 0.8	0.987
hsCRP ^2, 5^, mg/L	3.2 ± 2.6	2.9 ± 2.2	3.7 ± 2.5	3.0 ± 2.2	0.450
Glucose ^2^, mmol/L	5.5 ± 0.6	5.3 ± 0.4	5.4 ± 0.4	5.3 ± 0.4	0.341
Insulin ^2^, mU/L	12.4 ± 7.3	8.01 ± 4.2	13.4 ± 8.4	9.1 ± 4.5	0.334
SBP ^2^, mm Hg	120.6 ± 12.8	112.2 ± 9.7	119.4 ±12.9	112.2 ± 9.9	0.893
DBP ^2^, mm Hg	74.7 ± 9.0	71.1 ± 8.7	75.6 ± 9.4	71.5 ± 7.8	0.394

DER, standard meal replacement program; ADF + DER modified meal replacement program; BMI, body mass index; FFM, fat free mass; FM, Fat Mass; TC, Total Cholesterol; HDL-C, high density lipoprotein cholesterol; LDL-C, low density lipoprotein cholesterol; CRP, high sensitivity C Reactive Protein; SBP, Systolic blood pressure; DBP, Diastolic blood pressure. *p*-values are reported for between-group differences over time (time × diet interaction) using linear mixed-effects model analysis. ^1^ Values are Means ± SD. Biochemical measures, blood pressure and nutrient status assessed in fasting state. ^2^ significant main effect of time (*p* < 0.001), using linear mixed-effects model analysis. ^3^ significant effect of time × treatment (*p* < 0.05), using linear mixed-effects model analysis. ^4^ significant main effect of time (*p* < 0.01), using linear mixed-effects model analysis. ^5^ For CRP data, only participants with CRP concentration <10 mg/L were included in analyses; baseline: *n* = 71 DER; *n* = 68 ADF-MRP; week 16 *n* = 61 DER; *n* = 56 ADF + DER.

**Table 4:** Markers of nutritional status in the DER and ADF + DER groups before and after the 16-week energy restriction intervention ^1^.

	DER		ADF + DER		*p*
	Baseline (*n* = 81)	Week 16 (*n* = 68)	Baseline (*n* = 82)	Week 16 (*n* = 67)	
Iron, umol/L	16.2 ± 5.6	16.8 ± 5.5	15.1 ± 5.7	15.6 ± 5.5	0.921
Ferritin ^3^, µg/L	83.7 ± 66.9	90.8 ± 72.6	92.6 ± 96.5	114.6 ± 101.5	0.332
Transferrin ^2^, g/L	2.85 ± 0.4	2.7 ± 0.4	2.8 ± 0.4	2.7 ± 0.4	0.507
Transferrin Saturation, %	23.0 ± 8.8	25.1 ± 8.9	21.9 ± 8.7	23.7 ± 9.0	0.109
Serum Zinc ^4^, µmol/L	12.6 ± 1.5	13.0 ± 1.6	12.5 ± 1.5	12.8 ± 1.6	0.887
Folate ^2^, nmol/L packed red cells	1375.7 ± 290.2	1543.0 ± 318.8	1344.7 ± 274.7	1537 ± 286.8	0.533
Serum Vitamin B_12_^2^, pmol/L	315.2 ± 118.7	384.2 ± 145.1	287.8 ± 82.5	352.0 ± 101.4	0.664
Erythrocyte Thiamine Pyrophosphate ^2,^*, nmol/L	134.0 ± 22.6	121.9 ± 22.5	127.2 ± 20.7	114.1 ± 21.7	0.824
25 Hydroxy-Vitamin D ^3^, nmol/L	73.6 ± 20.4	71.7 ± 18.7	70.7 ± 17.4	70.7 ± 17.4	0.075

*p* values are reported for between-group differences over time (time × diet interaction) using linear mixed-effects model analysis. * Group difference at baseline (independent samples *t*-test), *p* < 0.05. ^1^ Values are Means ± SD. Biochemical measures, blood pressure, and nutrient status assessed in fasting state. DER, standard meal replacement program; ADF + DER modified meal replacement program; ^2^ significant main effect of time (*p* < 0.001), using linear mixed-effects model analysis. ^3^ significant main effect of time (*p* < 0.01), using linear mixed-effects model analysis. ^4^ significant main effect of time (*p* < 0.05), using linear mixed-effects model analysis.
